# Aerobic exercise and telomere length in patients with systolic heart failure: protocol study for a randomized controlled trial

**DOI:** 10.1186/s13063-022-06257-1

**Published:** 2022-04-11

**Authors:** Leandro T. Franzoni, Eduardo L. Garcia, Stephanie B. Motta, Mabel M. Ahner, Otávio A. Bertoletti, Marco A. L. Saffi, Anderson D. da Silveira, Alexandre A. Pereira, Adamastor H. Pereira, Luiz C. Danzmann, Ricardo Stein

**Affiliations:** 1 Post-Graduate Program in Sciences: Cardiology and Cardiovascular Sciences, Porto Alegre, Brazil; 2 Clinic Hospital of Porto Alegre, Porto Alegre, Brazil; 3 Federal University of Rio Grande do Sul, Porto Alegre, Brazil; 4 Porto Alegre, Brazil; 5 Lutheran University of Brazil, Porto Alegre, Brazil

**Keywords:** Exercise training, Cardiac rehabilitation, Heart failure, Functional capacity, Biological aging, Endothelium

## Abstract

**Background:**

Heart failure (HF) with reduced ejection fraction (HFrEF) is a syndrome that leads to fatigue and reduced functional capacity due to disease-related pathophysiological mechanisms. Aerobic exercise (AERO) plays a key role in improving HF outcomes, such as an increase in peak oxygen uptake (VO_2_peak). In addition, HF promotes cell senescence, which involves reducing telomere length. Several studies have shown that patients with a worse prognosis (i.e., reduced VO_2_ peak) also have shorter telomeres. However, the effects of AERO on telomere length in patients with HFrEF are still unknown. In an attempt to fill this gap, we designed a study to determine the effects of 16 weeks of aerobic training (32 sessions) on telomere length in HFrEF patients.

**Methods:**

In this single-center randomized controlled trial, men and women between 50 and 80 years old will be allocated into two different groups: a moderate-intensity aerobic training and a control grouTelomere length, functional capacity, echocardiographic variables, endothelial function, and walking ability will be assessed before and after the 16-week intervention period.

**Discussion:**

Understanding the role of physical exercise in biological aging in HFrEF patients is relevant. Due to cell senescence, these individuals have shown a shorter telomere length. AERO can delay biological aging according to a balance in oxidative stress through antioxidant action. Positive telomere length results are expected for the aerobic training group.

**Trial registration:**

ClinicalTrials.gov NCT03856736. Registered on February 27, 2019

## Background

HF affects more than 26 million people worldwide. It is considered a global public health problem and is expected to increase substantially with the aging of the population. Globally, HFrEF is the most prevalent form of HF syndrome, affecting at least 60% of all patients [1, 2]. Despite the different strategies for its management, most individuals with this syndrome will experience some limitation in exercise capacity during the natural course of the disease [3, 4]. In fact, exercise intolerance dominates the clinical presentation of moderate to severe HFrEF and is a major determinant of overall prognosis [5–7]. On the other hand, patients who exercise regularly have a better prognosis than sedentary ones [8], since AERO improves VO_2_ peak [9–11] and TL [12].

TL is a complex DNA sequence located at the ends of chromosomes [13–15]. It is important to point out that oxidative stress is the main factor that shortens TL in HFrEF [16–19] and accelerates the aging process [20–22]. Studies have shown that exercise can promote a reverse profile in oxidative stress, increasing TL or preventing telomere shortening [23–26]. However, changes in TL depend on exercise intensity. HIIT is described as short periods of exercise performed at a high intensity (> 80–85% heart rate reserve), with active recovery intervals at a moderate intensity (30–40% of HRR) [27]. MIAT (40–60% HRR), however, is the most commonly used AERO modality, and different HF guidelines recommended it [28–30]. Physiologically, very-high intensity exercise can lead to decreased TL due to an imbalance between severe oxidative stress and reduced antioxidant mechanisms [31, 32]. In contrast, MIAT can lead to a reduction in oxidative stress through higher antioxidant activity, which can have beneficial effects on TL [33–37].

In individuals who have not been diagnosed with HFrEF, conflicting results have been found regarding the effects of MIAT on TL. Some studies have shown that MIAT may increase TL [12, 24, 33, 34], while others have not observed any modification in these outcomes [38–40]. In patients with HFrEF, MIAT can improve functional capacity and has been demonstrated to be safe, effective, and reproducible outside the hospital environment [41–43]. However, as far as we know, no studies have investigated MIAT and TL in the HFrEF setting, and since there is a gap in the literature, the main goal of this manuscript is to describe the study protocol of this unique randomized controlled trial.

## Methods

We will compare TL in a MIAT group and a CG of HFrEF patients before and after 16 weeks of an exercise-based cardiac rehabilitation program. In addition, the secondary outcomes of this randomized controlled trial are to correlate TL with the following:

1. Different CPET parameters such as VE/VCO_2_, oxygen pulse, and oxygen uptake efficiency slope
2. Changes in echocardiographic variables by Doppler echocardiogram
3. Changes in endothelial function measured by FMD of the brachial artery
4. Changes in walking ability measured by SWSS

### Study design

This study will be a single-center randomized, controlled trial performed at a tertiary hospital in southern Brazil. Patients recruited to participate will be assigned to the MIAT group, who will exercise twice a week for 16 weeks, or the CG, who will stretch and do low-intensity and low-volume treadmill walking exercise (to mimic the intervention group) twice a week for 16 weeks. The public title for the work to patient recruitment is “Exercise to improve your heart and longevity.” Procedures explaining the intervention and the benefits of exercise will be applied in recruiting the patient

Measurements will be taken before and at the end of the follow-uFor familiarization, all subjects will participate in a run-in period involving three treadmill exercise sessions before randomization. The allocation ratio will be 1:1, and the framework will be superiority.

An experienced researcher in cardiac rehabilitation who is not involved in the data collection will apply the protocol. The study named “Exercise for improve your health” will be conducted at the hospital cardiac rehabilitation center with support from the CardioEx. The trial protocol was registered in ClinicalTrials.gov (identifier: NCT03856736) and follows the recommendations of the SPIRIT 2013 statement (Standard Protocol Items: Recommendations for Interventional Trials). The schedule of enrollment, interventions, and assessments is presented in Table 1.

**Table 1:**
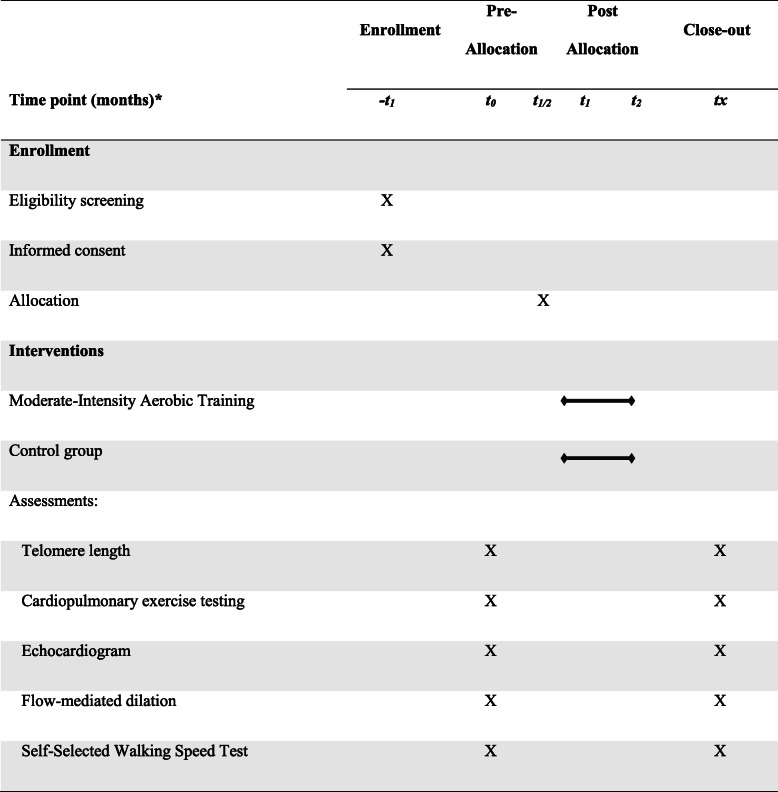
Schedule of enrollment, interventions, and assessments

*Note*: *t1, enrollment; *t0*, baseline assessment before randomization; *t1/2*, allocation; *t1*, start of interventions; *t2*, final assessment after interventions; *tx*, analysis of variables; *HF*, heart failure

### Participants

The volunteers will be recruited through the HF Outpatient Clinic of a tertiary public hospital in Porto Alegre, Brazil. Participants will be randomly allocated into two different groups: MIAT and the CG, which will engage in supervised low-intensity AERO with stretching.

#### Inclusion criteria

The following are the inclusion criteria:

- Primary diagnosis of HF with ejection fraction < 40%
- Clinically stable patients with at least 3 months on optimal HF treatment
- Age between 50 and 80 years
- NYHA functional classes II to III
- No contraindications to participate in an exercise program
- Mentally able to understand instructions during the study

#### Exclusion criteria

The following are the exclusion criteria:

- Severe valve disease
- Peripheral artery disease with symptoms of intermittent claudication
- Uncontrolled hypertension
- Drug or alcohol abuse
- Cognitive and/or osteomyoarticular conditions that prevent exercise
- Logistical impossibility of attending the hospital intervention
- Engaging in supervised physical exercise in the past 3 months
- Do not complete the run-in period

### Study procedures

**Fig. 1:**
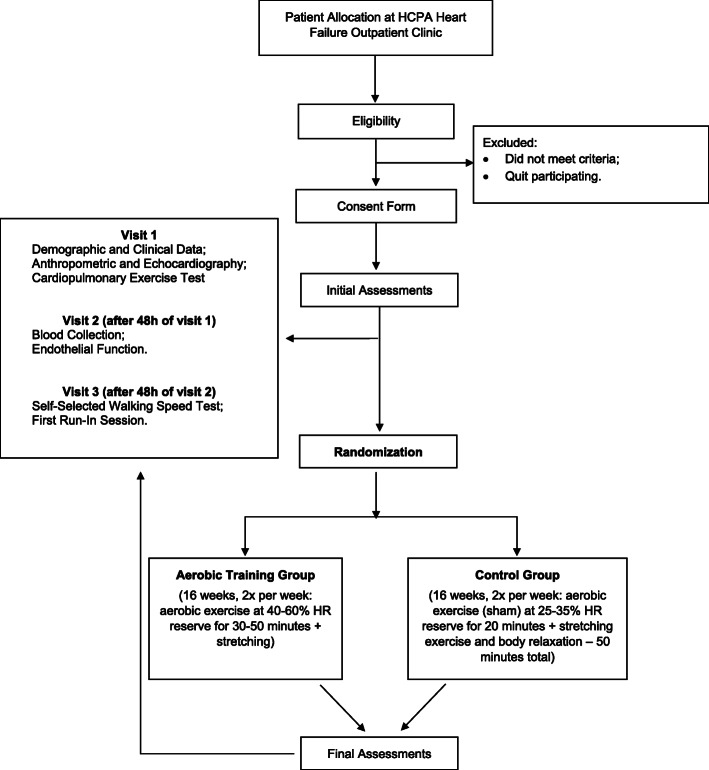
Study diagram

The protocol for both groups will be applied at a local tertiary hospital (HCPA). The study diagram can be seen in Fig. 1. After confirming the eligibility criteria during first contact, the researchers will obtain the written informed consent.

### Randomization

A researcher not involved in other phases of the study will perform the randomization and allocation protocols. The allocation list will be generated through the *randomization.com* website, and the data will be managed through the REDCap software in order to provide the allocation concealment. The randomization will be stratified by sex and age (50 to 64 years; 65 to 80 years), and different size blocks will be employed in a random order. Researchers involved in the data analysis and assessments will be blinded to the participant’s allocation group.

### Demographic and clinical variables

Demographic, anthropometric, and clinical data will be collected to characterize the sample. In addition, blood collection, CPET, echocardiography, endothelial function (assessed by brachial artery FMD measurement), and a SSWS test will be performed.

Age, gender, the presence of diabetes mellitus, systemic hypertension, and dyslipidemia will be some of the data used for sample characterization, as well as medical history and current medications. We will also measure waist, abdomen, and hip circumferences, as well as body mass and height, before participants begin the protocol.

### Telomere length

A real-time qPCR will be utilized to quantify TL. This technique is based on extending the telomere sequence from a small amount of genomic DNA.

In the present study, relative TL will be specifically evaluated, which is obtained through two qPCR reactions for each sample. One reaction is used for amplifying the T, while the other is for the S, which is responsible for controlling the amplification and allowing the number of genome copies per sample to be calculated. Therefore, the T/S ratio will be calculated to obtain a value that correlates with the average length of the analyzed telomeres [44, 45].

### Blood collection

The participants will rest for 15 min prior to blood collection. After the rest period, 10 mL of peripheral blood will be collected by trained personnel. The collected blood will be dispensed into 15-mL tubes containing EDTA anticoagulant and will be homogenized by inversion. Subsequently, the blood will be transferred to 15-mL tubes with a Histopaque® 1077 phase (density 1.077 g/mL, Sigma-Aldrich, St. Louis, MO, USA) at a 1:1 ratio and then centrifuged at 400×*g* for 30 min. Thereafter, centrifugation-purified peripheral blood mononuclear cells will be collected, from which genomic DNA will be extracted for subsequent qPCR.

### Cardiopulmonary exercise test

All evaluations will be performed during the morning shift at the HCPA noninvasive cardiology unit under controlled temperature (18 to 22 °C). The tests will always be performed by the same cardiologist, who is qualified by the Brazilian Society of Cardiology. The test will be performed on a treadmill (General Eletric T-2100, GE Healthcare, Waukesha, WI, USA) using a ramp protocol previously described in Nery et al. [46] VO_2_, VCO_2_, ventilatory anaerobic threshold, respiratory compensation point, peak respiratory exchange ratio, VE/VCO_2_ slope, oxygen uptake efficiency slope, and O_2_/HR will be measured and recorded breath by breath with a specific CPET system for measuring pulmonary gas exchange (Quark CPET, COSMED, Rome, Italy). Continuous 12-lead electrocardiographic monitoring (Nihon Kohden Corporation, Tokyo, Japan) will be performed following Mason and Likar 1966. Blood pressure measurement will be assessed with a sphygmomanometer (P.A. MED PA 2001, Brazil). Maximum tests will be considered when the peak respiratory exchange ratio is ≥ 1.05.

### Transthoracic Doppler echocardiogram

All evaluations will be performed by a trained cardiologist on the same equipment at the HCPA noninvasive medicine unit (Envisor C HD, Philips, USA) with a standard multifrequency sector transducer. Patients will be evaluated at rest in the left lateral supine position. Ultrasound equipment will be placed on the patient’s chest and the signals will be transmitted and converted into a moving image on a monitor. Subsequently, the diameters and volumes of the atrium and left ventricle will be measured. The ejection fraction will be calculated using the Teicholz formula from the parasternal long axis. However, for patients with regional wall motion abnormalities, Simpson’s rule will be used. The assessment will proceed according to the current guidelines of the American College of Cardiology and the American Heart Association [47].

### Endothelial function

The assessments will be performed according to the recent expert consensus and evidence-based recommendations on flow-mediated dilatation in humans [48]. The volunteers will receive preparation instructions, such as the need to fast for 6 h prior to evaluation; no smoking or tobacco consumption prior to measurement (> 6 h); avoiding exercise (> 24 h), caffeine, and alcohol (> 12 h) prior to the evaluation; recording medication used in the 24 h prior to assessment; and premenopausal women should record the day of the menstrual cycle, since the evaluation will be between the first and seventh cycle day.

The pre- and post-assessment will be performed at the same hour, in a room with a controlled temperature (18 to 22 °C). The volunteers will have 10–15 min of supine rest prior to beginning their assessment. During the assessment, the volunteers will be asked to lie in the supine position with their left arm positioned comfortably. Endothelium-dependent and endothelium-independent dilations will be measured by spectral Doppler ultrasound (Ultrasonix, Ultrasonix Medical Corporation, Richmond, Canada) with a modulated electrocardiogram and a high-frequency vascular transducer (between 7.5 and 14 MHz). FMD will be expressed as the relative variation of the brachial diameter in the hyperemic phase and defined as [(post hyperemic diameter − baseline diameter)/baseline diameter] × 100.

### Self-selected walking speed

This test will be performed during the first training session to determine the volunteers’ SSWS. The test will be conducted in a 30-m corridor, demarcated every 3 m with cones, as previously described by Monteiro et al [49].

To balance any effects related to the participant’s sensation of being evaluated and wanting to walk faster, timing will begin not with the first cone but the second. Since this test is a measure of self-selected speed, we must ensure that it is performed with no stimulus to walk faster, especially when the subject is approaching the final cone. However, the timer will be stopped prior to the final cone for the same reasons as the first cone. Therefore, the evaluation will consist of the time taken to walk 24 m. To calculate the SSWS, the distance traveled will be divided by the time necessary to do so; three attempts will be performed, and the mean time will be considered the SSWS.

### Intervention protocols

**Fig. 2:**
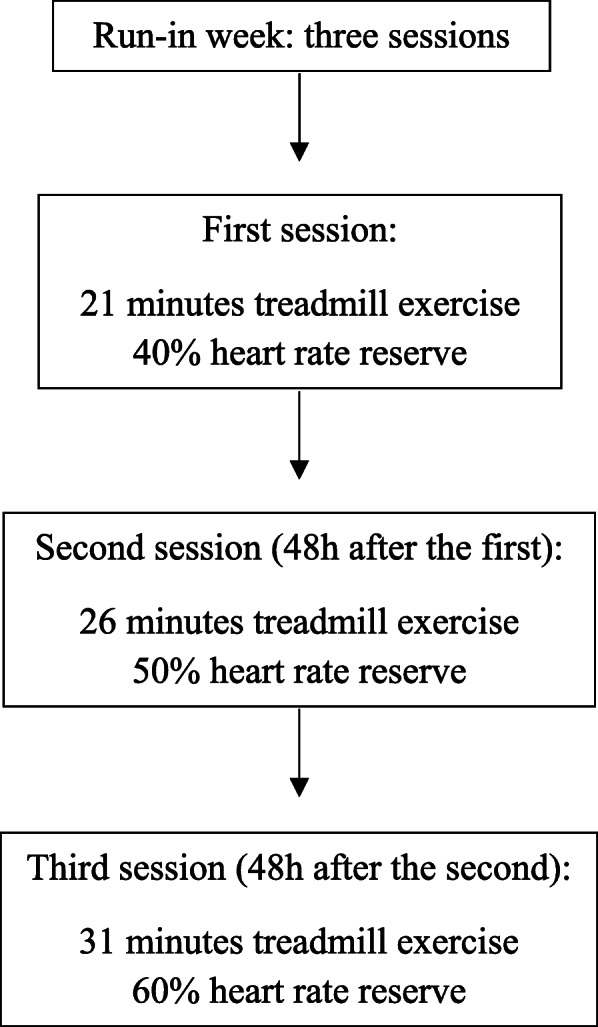
First week run-in period

The aerobic training model will follow a predefined schedule (Table 2). The first week will be a run-in period (Fig. 2), consisting of three sessions of moderate-intensity AERO with a progressive increase in session duration. Both groups will perform treadmill exercise and stretching. However, the intensity and duration of treadmill walking and stretching will change. Because this will be a blind randomized clinical trial, we must ensure that the volunteers do not know the group in which they will be participating. Both protocols will last 16 weeks and involve sessions twice a week, totaling 32 sessions for each grouThe minimum frequency will be 85% of the total sessions. In the event of three consecutive absences, the volunteer will be excluded from the study, as well as if the patient appears only one session per week for three consecutive weeks. To improve adherence, we will use daily motivation in each session, such as praise, reminders that the treatment is good for him/her, or that the patient is doing well and this will make a lot of difference to their health, regardless of which group was randomized (exercise or control).

**Table 2:** Predefined periodization for the aerobic training group

Intervention period	Exercise prescription
Weeks 1–2	Time 30–35 min—40–45% HRR = equivalent % VO_2_peak
Weeks 2–3	Time 35–40 min—45–50% HRR = equivalent % VO_2_peak
Weeks 3–4	Time 35–40 min—50–55% HRR = equivalent % VO_2_peak
Weeks 4–6	Time 40–45 min—55–60% HRR = equivalent % VO_2_peak
Weeks 6–7	Time 45–50 min—50–60% HRR = equivalent % VO_2_peak
Weeks 7–8	Time 45–50 min—50–60% HRR = equivalent % VO_2_peak
Weeks 8–9	Time 45–50 min—50–60% HRR = equivalent % VO_2_peak
Weeks 9–11	Time 45–50 min—50–60% HRR = equivalent % VO_2_peak
Weeks 11–12	Time 45–50 min—50–60% HRR = equivalent % VO_2_peak
Weeks 12–13	Time 45–50 min—50–60% HRR = equivalent % VO_2_peak
Weeks 13–14	Time 45–50 min—50–60% HRR = equivalent % VO_2_peak
Weeks 14–15	Time 45–50 min—50–60% HRR = equivalent % VO_2_peak
Weeks 15–16	Time 45–50 min—50–60% HRR = equivalent % VO_2_peak

*HRR*, heart rate reserve

The training prescription will be individualized according to CPET. The target training zones will be definsssssed through percentages of heart rate reserve and ventilatory thresholds, concomitant with the use of a modified Borg CR10 scale [50] at moderate intensity (40–60% of heart rate reserve; Borg 4–6). The protocol will begin with a warm-up and will have a cool-down period. Variables such as speed and grade will be systematically adjusted each week according to each patient’s chronotropic response, effort perception, and condition. When necessary, it will be returned to the previous level until the individual adapts and can progress.

Exercise sessions will be prescribed and accompanied by an exercise physiologist who may be accompanied by undergraduate physical education, physical therapy, or medicine students who will monitor and record HR, blood pressure, and perceived exertion before, during, and after the exercise session. The training program will be performed on treadmills (Inbramed, Export, Porto Alegre, Brazil, and TEB APEX 2000, São Paulo, Brazil).

### Control group

The CG will undergo two sessions weekly, totaling 32 sessions. Stretching exercises, low-intensity treadmill walking, and body relaxation techniques will be performed. Given the literature gap about the effects of AERO on TL in HF patients, we decided to create a CG with the same intervention time and weekly frequency that performs low-intensity AERO on a treadmill to mimic the intervention group and consequently investigate the real effects of MIAT advocated in different guidelines, with all participants blinded as far as possible to intervention type.

### Statistical analysis

The calculated sample size is 10 patients for each group (20 total), considering a significance level of 5%, a power of 80%, a difference to be detected equal to a standard deviation of 0.0026 for TL, and the primary outcome. Based on other studies, we estimate there will be a 20% loss, so it will be necessary to include 12 patients per group (24 patients in total). The difference to be detected is considered clinically relevant, and the variability was based on Van der Harst et al. [16]

Descriptive statistics will be performed with mean and standard deviation or median and interquartile range when appropriate. The Shapiro-Wilk test will be performed to verify data normality. Baseline sample characteristics will be compared using Student’s *t* test or the Mann-Whitney *U* test for continuous variables and the chi-square and Fisher exact test for categorical variables. The outcomes for the MIAT and the CG during the pre- and post-training periods will be analyzed with generalized estimating equations. A Bonferroni post hoc test will be used to identify the differences between the effects and interactions. Intention to treat will be applied

## Discussion

Telomere and its length have been studied as a biological marker of aging and are considered a therapeutic target, not only in patients, but also in healthy individuals [51, 52]. The larger the telomere, the greater the life expectancy of the individual [53].

Acute AERO can promote the upregulation of telomeres and the expression of white blood cell microRNAs, improving immune function and physical health [54]. In its turn, chronic physical training plays an important role in maintaining or increasing the TL [23]. Some evidence suggests that only AERO (moderate or high intensity) can increase the TL after 6 months of intervention in healthy individuals [12] and there is already some evidence that MIAT can have a positive impact on TL in some pathological scenarios [33–37].

HFrEF patients present a decrease in VO_2_ peak and an increase in oxidative stress, findings that go towards a more reserved prognosis. It is important to point out that the worse the disease, the shorter the telomeres [23]. In contrast, MIAT can increase VO_2_ peak and reduce oxidative stress through greater antioxidant activity, acting positively on TL. However, specifically, the effect of MIAT on TL in the HFrEF scenario is unknown, but we will test if 6 months of this type of training can delay biological aging, promoting a positive impact on TL in these stable patients with this syndrome.

## Data Availability

Data sharing is not applicable to this article as no datasets were generated or analyzed during the current study. Relevant data from this study will be made available upon study completion and researcher’s request from the corresponding author.

## Abbreviations

HF: Heart failure
HFrEF: Heart failure with reduced ejection fraction
AERO: Aerobic exercise
VO_2_peak: Peak oxygen uptake
TL: Telomere length
HIIT: High-intensity interval training
HRR: Heart rate reserve
MIAT: Moderate-intensity aerobic training
CG: Control group
CPET: Cardiopulmonary exercise testing
VE/VCO_2_: Ratio between ventilation and the carbon dioxide production
FMD: Flow-mediated dilation
SWSS: Self-selected walking speed test
CardioEx: Exercise Cardiology Research Group
NYHA: New York Heart Association
HCPA: Hospital de Clínicas de Porto Alegre
REDCap: Research Electronic Data Capture
qPCR: Polymerase chain reaction
T: Telomeric sequence
S: Single-copy gene
VO_2_: Oxygen uptake
O_2_/HR: Oxygen pulse
